# Chronic impalement- Case report of a knitting needle 33 years’ ascension

**DOI:** 10.1186/s13019-016-0511-2

**Published:** 2016-07-29

**Authors:** Marian Pop, Bogdan Andrei Suciu

**Affiliations:** 1 Doctoral school, University of Medicine and Pharmacy of Tîrgu Mureş, Gh. Marinescu 38, Tîrgu Mureş, 540139 Romania; 2 Radiology and Medical Imaging Laboratory, Emergency Institute for Cardiovascular Diseases and Transplantation (IUBCvT) Tîrgu Mureş, Gh. Marinescu 50, Tîrgu Mureş, 540136 Romania; 3 Anatomy Department, University of Medicine and Pharmacy of Tîrgu Mureş, Gh. Marinescu 38, Tîrgu Mureş, 540139 Romania; 4 First surgery clinic, Tîrgu Mureş Emergency Clinical County Hospital, Gh. Marinescu 50, Tîrgu Mureş, 540136 Romania

**Keywords:** Foreign body, Thorax, Computed tomography, Chest x-ray, Case report

## Abstract

**Background:**

We report the case of a 72-year-old female complaining of hemoptysis due to a thoracic-abdominal knitting needle inserted 33 years ago for self-induced abortion.

**Case Presentation:**

The PA/LL chest x-ray showed a metallic foreign body on thorax extending into the abdomen. An CT examination confirmed the transdiaphragmatic knitting needle extending from liver into right upper lobe.

**Conclusions:**

Surgical removal of the foreign body and wedge resection were performed with good follow-up results.

## Background

The sources of foreign bodies in adults are more frequently iatrogenic or traumatic [7].

This is a report of a patient with a metallic foreign body (knitting needle) inserted for self-induced abortion found 33 years later extending from the liver segment VIII into right upper pulmonary lobe.

Abdominal knitting needles used for unsafe abortions are described in literature [1, 10] but finding an abdominal knitting needle in a thoracic location is rare [3–5, 11]. Data from Schechter [13] present thoracic knitting needles as cause for 2.72 % of all thoracic injuries due to needles. Migrating thoracic foreign body have been described [6] but there is no literature about one’s movement from pelvis to thorax.

## Case presentation

A 72 years old female complaining of hemoptysis, dyspnea, fatigability and weight loss was referred to the surgery clinic. A history of 7 abortions was obtained. 33 years ago she self-induces an abortion using a knitting needle without extraction of the foreign body.

Clinical exam showed a decreased mobility of the right hemidiaphragm but no scars. Hemoglobin and hematocrit were borderline low (HGB 11.6 and HCT 38.4), with RBC indices suggestive for anemic status (MCV 67.5, MCH 20.4). Pulmonary function tests showed decreased forced vital capacity (FEV) and Forced expiratory volume in 1 s (FEV1).

**Fig. 1:**
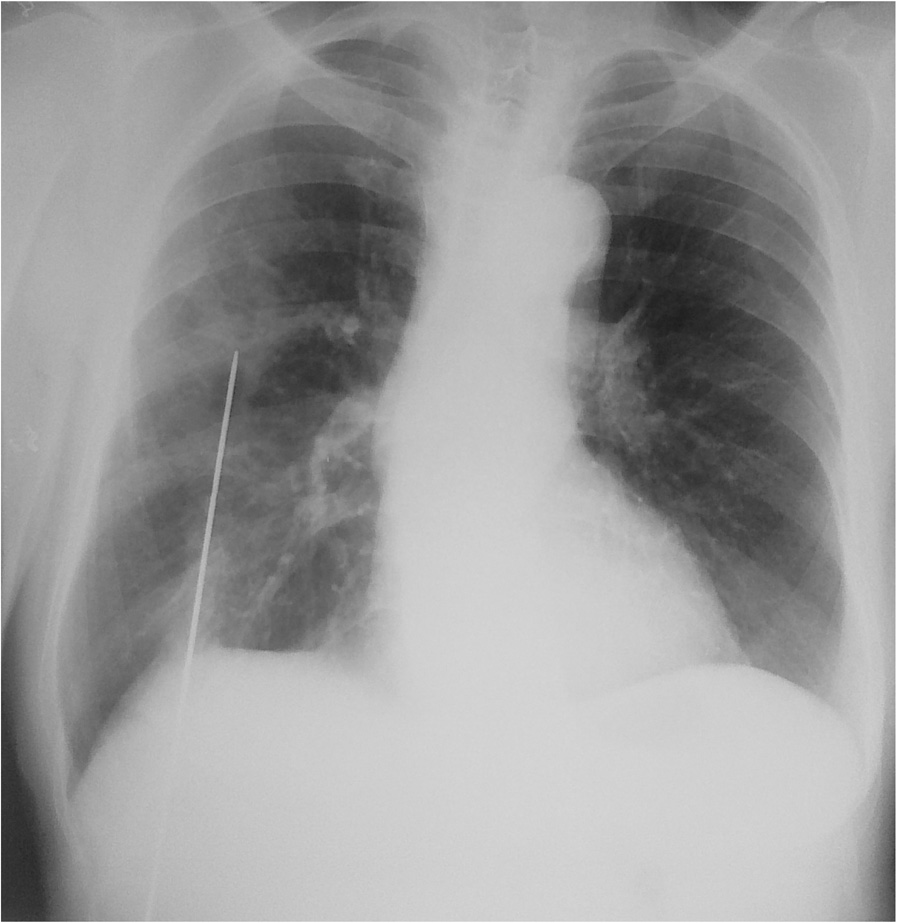
Initial chest X-ray showing a metallic foreign body extending from abdomen into chest

**Fig. 2:**
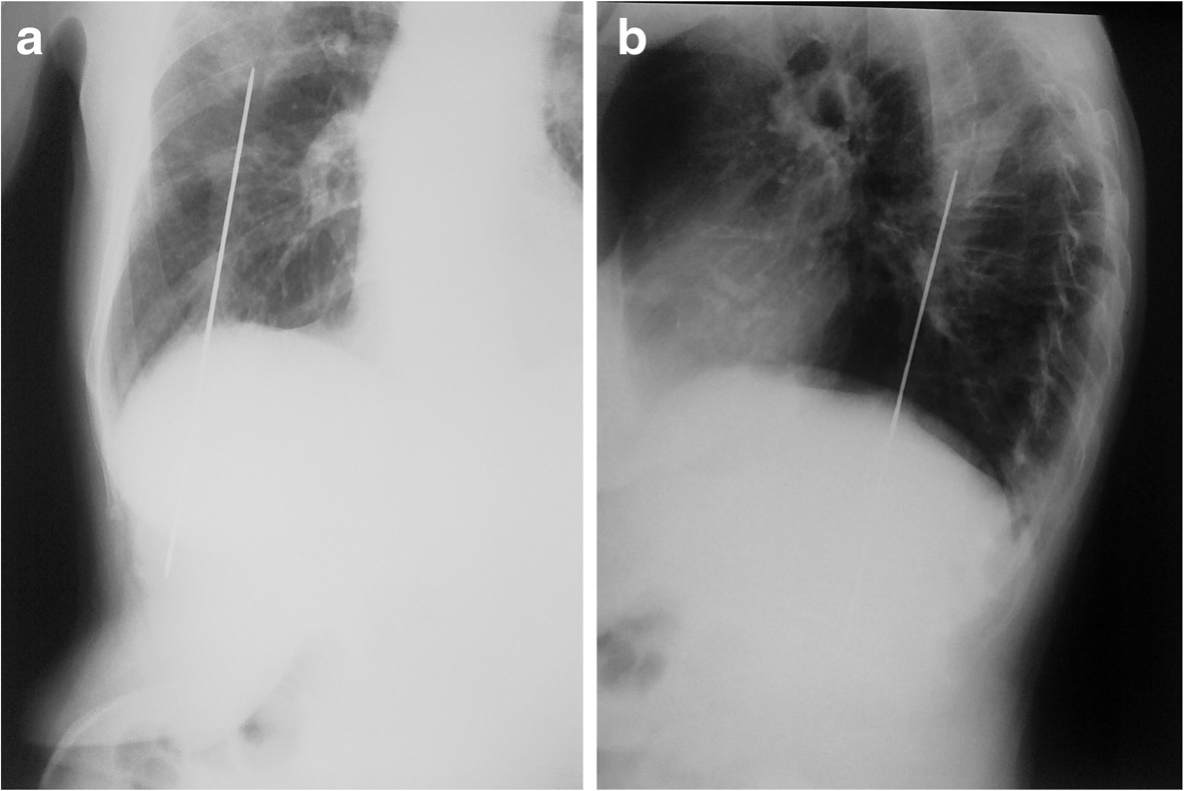
**a** and **b** Follow-up X-rays (PA and lateral) focused on the foreign body demonstrating its location as thoraco-abdominal

Initial radiological workup included a chest x-ray which showed a linear metallic foreign body extending from mid-thorax into the abdomen (Fig. 1). Two more x-rays were performed (Fig. 2a and b), one focused on the lesion and another one in lateral view, establishing its caudal end at the level of the liver and its size at 18 cm. In retrospect a focal increased density lesion is viewed in the posterior-basal segment of right inferior lobe.

**Fig. 3:**
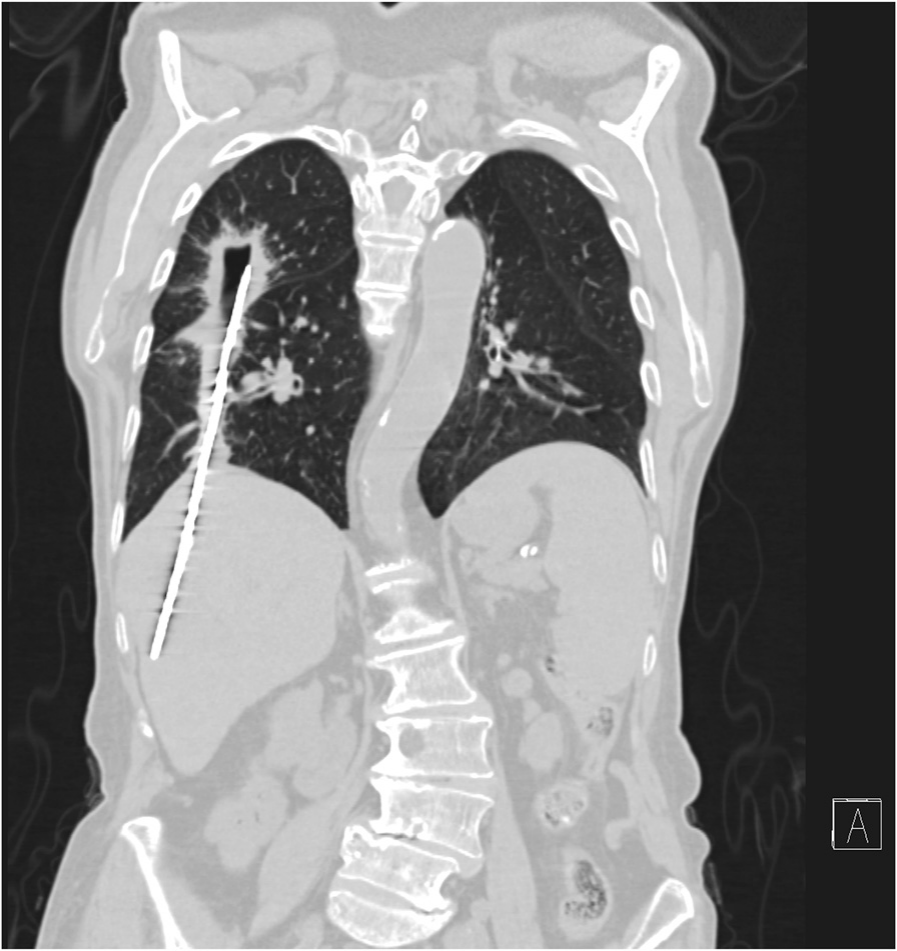
Coronal slice of thoraco-abdominal CT. There is an 18 cm metallic foreign body extending from right liver lobe (segment V and VIII), transdiaphragmatic, to the right upper lobe

**Fig. 4:**
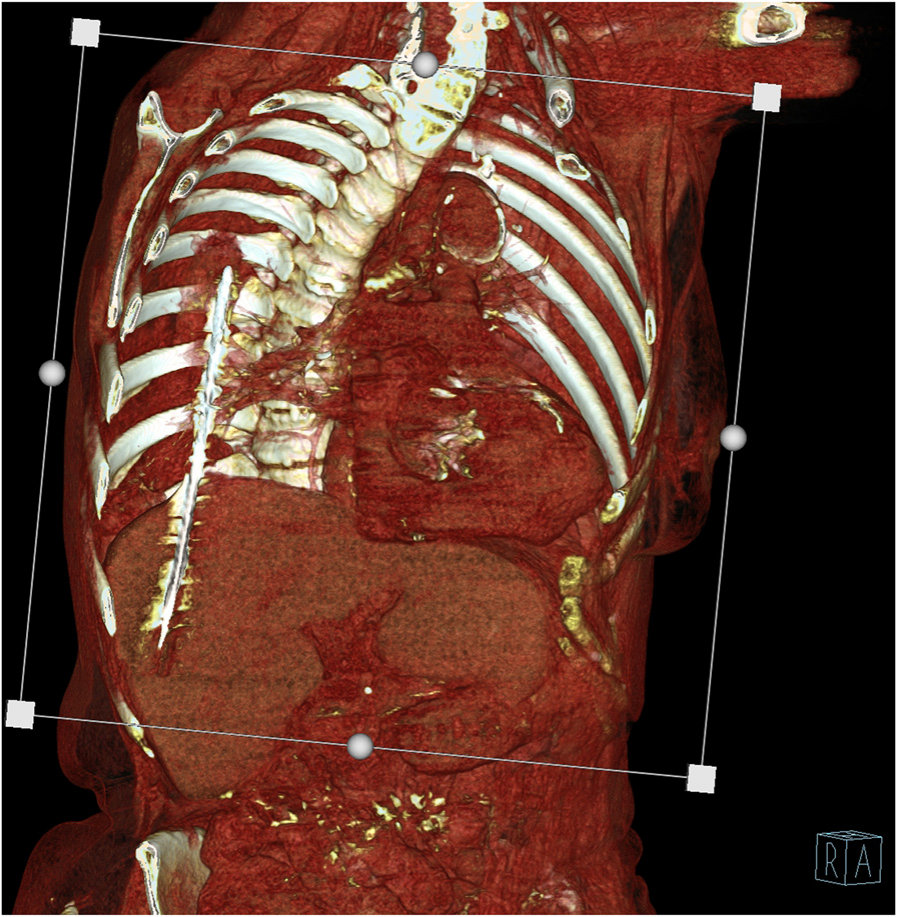
CT examination- VRT reconstruction demonstrating the foreign body

Following clinical meeting a CT was ordered showing the foreign body extending from right liver lobe (segment V and VIII), transdiaphragmatic, to the right upper lobe, where it ends in a thick walled cavity of 3 cm (Figs. 3 and 4). No traces of migration have been described in the abdomen or pelvis. A 3.9 cm consolidation in the posterior-basal segment of RLL was also described with associated mediastinal adenopathy. No abdominal or bone lesions were detected.

**Fig. 5:**
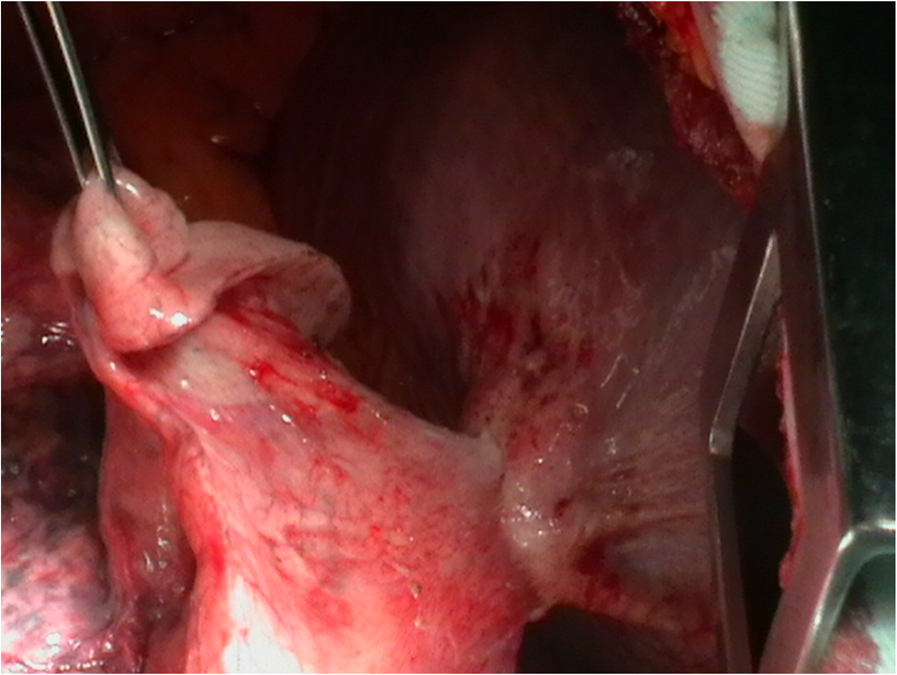
Intraoperative photograph of right posterolateral thoracothomy. Right Lower Lobe and diaphragm are visualized, with fibrous tissue surrounding the foreign body

**Fig. 6:**
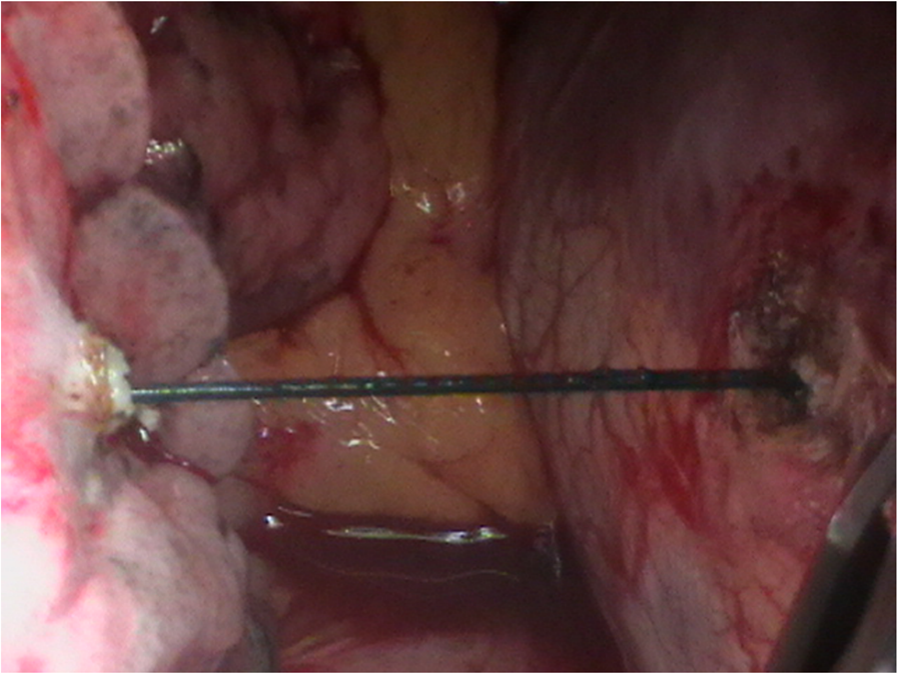
Intraoperative aspect after fistula closure. The transdiaphragmatic foreign body is visualized

Right posterolateral thoracothomy was performed. Intraoperative examination detected pulmonary right lower lobe to diaphragm adhesions and fibrous tissue delimiting a thoracoabdominal transdiaphragmatic fistula due to a foreign body (Fig. 5). An 18 cm metallic foreign body producing a hepatic fistula of 4 cm and RLL fistula of 8 cm is extracted (Fig. 6). Wedge resection of RLL and diaphragmatic mesh suture with infradiaphragmatic drainage were performed.

The postoperative results were good, with the patient discharge 8 days later. Only one follow-up was available; at 3 months’ control the patient was in good status with no complains.

## Discussion

Thoracic foreign bodies can be found as a result of accident or traumatic event. Although usually there is evidence of penetrating trauma, significant internal injuries may occur without noticeable external injuries or scars. Penetrating wounds of the thorax may induce pneumothorax (in 20 % of cases) or hemothorax (up to 80 %) [7].

An injury penetrating into the lung may be backed by clinical and imaging findings.

Consideration should be paid to the personal history, mechanisms of injury and radiologic findings. Various kinds of foreign bodies have been reported in the radiologic literature, with metallic and other high-attenuation foreign bodies easily being detected radiological means [7].

Migration of iatrogenic foreign bodies has been described (with effects varying from minimal [12] to catastrophic [8]) but because the clinical follow-up they usually benefit from early detection and they will not move for decades.

The unsafe abortion carried out either by persons lacking the necessary skills or in an environment that does not conform to minimal medical standards [14] represent 56 % of the induced abortions, with an increase from 44 % in 1995 to 49 % in 2008 [14]. Involving the insertion of a solid object into the uterus, the unsafe abortion may be induced by the woman herself or by a nonmedical person, with high mortality and morbidity risks [2].

Our case was a fortunate one for the patient. It is likely that the original puncture was through a relatively less vascular part of the uterine wall which would have caused abdominal pain and bleeding per vaginum but was assumed to be due to abortion, as in other cases of unsafe abortion [9]. While the mechanism of migration remains an enigma, probably following uterine involution, myometrial contractions and musculature movement the needle was pushed for 3 decades into its current position.

## Conclusion

This report shows the case of a foreign body (knitting needle) which, being retained after an unsafe abortion started moving for 33 years through the body of a woman, being found and surgically removed from a thoracoabdominal site.

## Abbreviations

FEV, forced expiratory volume; FEV1, forced expiratory volume in 1 s; HCT, hematocrit; HGB, hemoglobin; MCH, mean corpuscular hemoglobin; MCV, mean corpuscular volume; RBC, red blood cells count; RLL, right lower lobe
